# Mental Hospitals and Recruiting

**Published:** 1915-07-31

**Authors:** George M. Robertson

**Affiliations:** Physician Superintendent. The Royal Asylum, Morningside, Edinburgh.


### THE EDITOR'S LETTER-BOX.

### Mental Hospitals and Recruiting.

*To the Editor of* The Hospital.

Sir,—The male attendants of military age employed in asylums have, as a class, not been backward in their duty to their country. Many of them enlisted at the beginning of the war, being encouraged to do so by the loyal attitude of asylum boards and committees as regards pay, etc., and the vacancies created by these absentees have since been largely filled by men who are ineligible for military service. There are still, however, at the present time very many able-bodied young men employed as attendants on the insane. The presence of some of these is necessary, but the number required can be considerably reduced by the substitution of female nurses, especially in place of those attendants engaged in nursing the sick in the male hospitals and male infirm wards of asylums.

Fifteen years have now elapsed since the experiment of employing women on a large scale to nurse male patients in an asylum was tried in Scotland. All that was then said as to the advantages of female nursing for insane men under certain limitations has since been proved true by many observers, and the fears of the early opponents of this system have been found to be negligible, for the dangers that they prophesied have, by forethought and care, been avoided. The system is now, from the practical experience they have gained of its benefits, very strongly advocated by the Scottish Board of Control, and their judgment can be accepted without reserve. It has been all but universally adopted by the Scottish asylums.

As there is every indication that all eligible males who can be spared will soon be wanted for our military forces, I take this opportunity of directing the attention of the medical superintendents and the members of boards and committees of asylums in England to this system, as it has up till now scarcely been introduced into English asylums. Not only will they be doing a patriotic duty by introducing these female nurses to enable more of their male attendants to enlist, but they can be assured that they will at the same time be adding to the comfort and well-being of the sick and infirm male patients under their charge.—I am, yours faithfully,

George M. Robertson, M.D.,
Physician Superintendent.

The Royal Asylum,
Morningside, Edinburgh.
July 21, 1915.

